# Thermal nociceptive properties of trigeminal afferent neurons in rats

**DOI:** 10.1186/1744-8069-6-39

**Published:** 2010-07-07

**Authors:** Jason M Cuellar, Neil A Manering, Mikhail Klukinov, Michael I Nemenov, David C Yeomans

**Affiliations:** 1 Department of Anesthesia, Stanford University School of Medicine, 300 Pasteur Drive, Stanford, California 94305, USA; 2 Lasmed LLC, 137 Irene Ct, Mountain View, California 94043, USA

## Abstract

**Background:**

Although nociceptive afferents innervating the body have been heavily studied form many years, much less attention has been paid to trigeminal afferent biology. In particular, very little is known concerning trigeminal nociceptor responses to heat, and almost nothing in the rat. This study uses a highly controlled and reproducible diode laser stimulator to investigate the activation of trigeminal afferents to noxious skin heating.

**Results:**

The results of this experiment demonstrate that trigeminal thermonociceptors are distinct from themonociceptors innervating the limbs. Trigeminal nociceptors have considerably slower action potential conduction velocities and lower temperature thresholds than somatic afferent neurons. On the other hand, nociceptors innervating both tissue areas separate into those that respond to short pulse, high rate skin heating and those that respond to long pulse, low rate skin heating.

**Conclusions:**

This paper provides the first description in the literature of the in vivo properties of thermonociceptors in rats. These finding of two separate populations aligns with the separation between C and A-delta thermonociceptors innervating the paw, but have significant differences in terms of temperature threshold and average conduction velocities. An understanding of the temperature response properties of afferent neurons innervating the paw skin have been critical in many mechanistic discoveries, some leading to new pain therapies. A clear understanding of trigeminal nociceptors may be similarly useful in the investigation of trigeminal pain mechanisms and potential therapies.

## Background

Trigeminal afferent neurons relay sensory and proprioceptive information from orofacial regions and intracranial meninges. Despite evident analogies with the spinal sensory system, accumulating data are in favor of fundamental anatomic, functional and trophic factor requirement peculiarities of the trigeminal sensory system [1-3]. Together with the functional complexity of trigeminal sensory systems, this specificity might account, at least in part, for the real therapeutic challenge presented by trigeminal pain. However, unlike the spinal (body) nociceptive system, there is very little known about the characteristics of mammalian trigeminal primary afferents *in situ*. In particular, although some authors have described responsivity of mechanoceptors [3] and meningeal afferent neurons [4], only Beitel and Dubner [5,6] have described thermoresponsiveness of trigeminal afferent neurons. The experiments described in these papers, which were performed in monkeys, used a Peltier thermode to provide the only extant descriptive information concerning receptive field, temperature thresholds, mechanical sensitivity and conduction velocity. However, this groundbreaking work did not investigate responses of myelinated (A-delta) fiber thermonociceptors, limiting their work to examining trigeminal unmyelinated (C) fiber nociceptors.

Different classes of nociceptors may be important in different types of clinical pain. For example, in post-surgical patients, the ongoing aching pain at the incision site is likely mediated by unmyelinated nociceptors; the acute sharp pain evoked by movement of the wound by dressing change, ambulation, coughing, etc., is likely mediated predominantly by myelinated (A-delta) nociceptors [7]. This distinction appears to be mediated, at least in part, by the rapid accommodation to a constant stimulus by A-delta fiber nociceptors, and the lack of this characteristic by C nociceptors [8]. Thus, the differential physiology of these nociceptor types dictates the characteristics of the pain experienced. Similarly the differential receptor and channel population of these different nociceptor types also dictates the modulation of their responsiveness by endogenous or exogenous chemicals. For example, C fiber nociceptors appear to possess mu opioid receptors; A-delta fiber nociceptors appear not to [9]. This may explain the greater efficacy of morphine on the ongoing, aching pain after surgery and the impotency of morphine on the acute, sharp pain accompanying dressing changes, etc [7]. Thus, different nociceptor types possess differentiable activation physiology and modulation pharmacology.

Previous work from our laboratory as well as others has suggested that varying the characteristics of noxious skin heating may allow for controlled differential activation of myelinated or unmyelinated cutaneous nociceptors [10-18]. As early as 1952 however, the inherent contamination of contact thermal stimuli was recognized [19]. Many studies have used radiant skin heating, generated by a projector or infrared bulb, as a source of heat stimulation, for example in the tail-flick and plantar behavioral thermonociceptive assays. While an improvement, the output variability, gradual degradation over time, and non-specific output spectrum were all recognized as limitations of this technology. Thus, laser stimulators have been developed which provide uniform, monochromatic light, allowing for rapid, flexible, and precise skin heating. Most experimental work to date has used either CO_2 _or Thulium lasers [20-30]. However, we have focused on using diode infrared laser irradiation, which provides extremely stable, monochromatic infrared light, which, in turn, allows for relatively uniform heating at depth in the skin. Because of the inherent penetration of near infrared wavelengths, this method allows for direct heating of cutaneous nociceptor terminals, obviating the necessity of overheating the surface in order to provide sufficient heat to activate sub-surface receptors.

We have performed behavioral experiments examining the differential effects of short and long infrared laser pulses on trigeminal nociception [31]. This experiment demonstrated that brief (200 ms), high intensity pulses applied to a 2 mm diameter spot on the face of rats evoked a rapid withdrawal response that is intensity (laser power) dependent. These responses are not affected by topical capsaicin or injection of 1 mg/kg morphine, but are sensitized by topical DMSO, characteristics that suggest A-delta fiber mediation [13,31-33]. On the other hand, a long duration pulse (10-20s), applied over a 5 mm spot size evoked withdrawal responses that were sensitized by topical capsaicin, were attenuated by 1 mg/kg of morphine and were unaffected by topical DMSO, providing evidence for mediation by C fiber nociceptor activation. There results provide presumptive evidence of differential nociceptor mediation of behavioral responses to high rate vs. low rate laser skin heating. The current study utilized *in vivo *single-unit trigeminal ganglia recording experiments performed to provide direct determination of nociceptor activation under different laser irradiation conditions. In addition, to accurately assess skin temperature changes evoking responses, particularly with high heating rates, intense brief pulses were used and a high-speed thermal camera was employed in some experiments. This study therefore directly investigated the differential activation characteristics of A-delta or C thermonociceptor types using infrared diode laser stimulation of trigeminal nerve-innervated facial skin in rats.

## Results

Recordings were performed at an average of 2.61 mm lateral (range: 2.25 mm-3 mm) and 1.5 mm caudal (range: 1.5 mm-1.5 mm) to bregma. The average recording depth (distance from brain surface) was 9.73 mm (range: 9.09 mm-10.65 mm)

**Figure 1:**
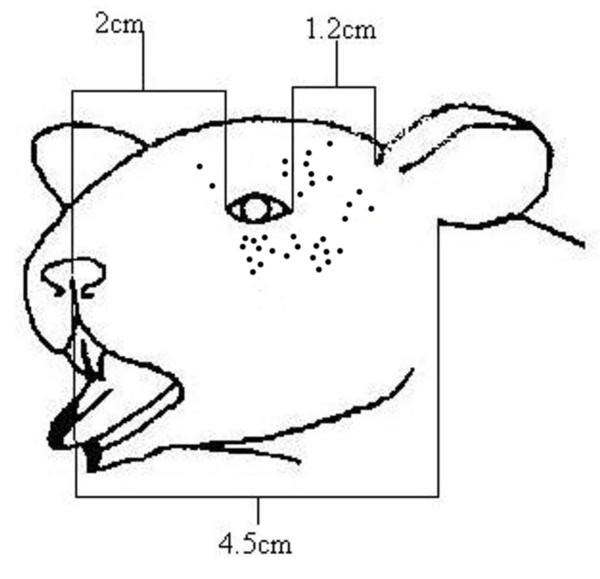
**Receptive fields of recorded thermonociceptors**. Receptive fields, first identified using mechanical stimuli, of all afferents recorded are shown as black dots near the eye.

All recorded neurons were sensitive to mechanical pinch with which receptive field areas were initially determined. The RFAs of the face were of fairly uniform size and were located in the regions innervated by the ophthalmic and maxillary branches of the trigeminal nerve (V1 and V2, respectively) [34]. In particular, units were concentrated within 12 mm of the eye in the ventral, ventrocaudal, caudal, and dorsocaudal directions (see Figure 1).

### Afferent Conduction Velocity and Intensity Response

**Figure 2:**
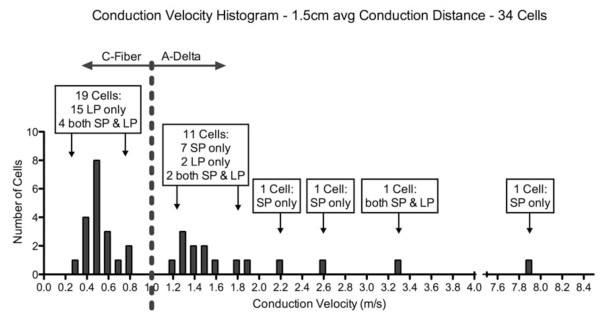
**Distribution of Conduction Velocities of Recorded Neurons**. Number of afferents along the spectrum of conduction velocities, determined using delay to response to electrocutaneous stimulation of receptive fields are shown, along with the response specificity of those afferents.

**Figure 3:**
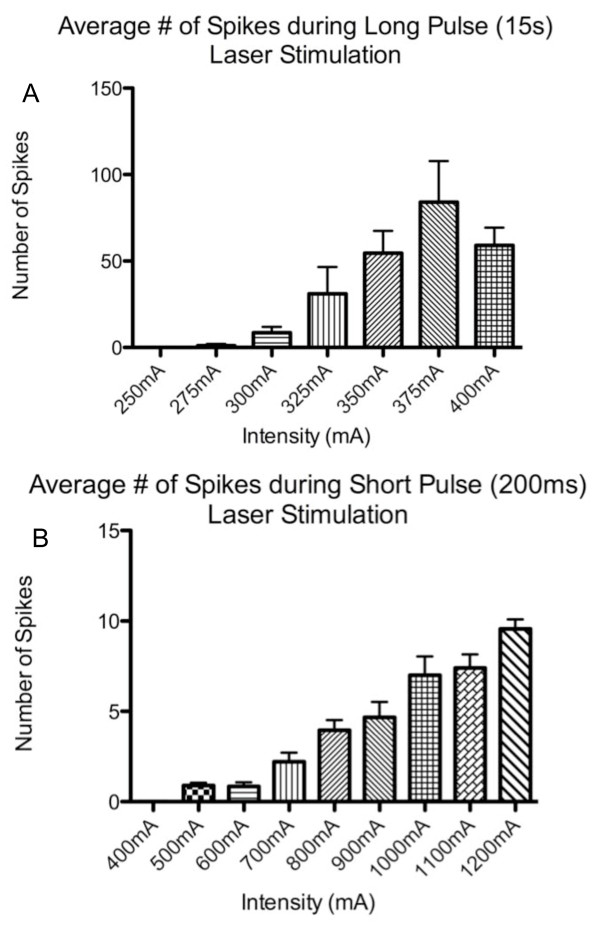
**Intensity Response Distribution**. Afferent neurons demonstrated a relatively linear increase in number of action potentials ("spikes") evoked in response to increasing laser stimulus intensity. This observation was true both for short, high power and long, lower power laser diode stimuli.

Based on nerve conduction velocity (CV) measures, we observed two distinct but overlapping populations of thermonociceptors (see Figure 2). Units with CV < 1.2 m/s were classified as C-fiber mechanoheat nociceptors (CMH). Units with CV ≥ 1.2 m/s were classified as A-delta-mechanoheat (AMH) nociceptors. The length from the center of the trigeminal ganglion to its point of innervation just ventral to the eye was measured and found to average 15 ± 1 mm in the four rats measured. Conduction distance for other animals was inferred from these measurements. Based on the conduction distance, out of 34 units, 19 C fiber afferents conducted very slowly within a range of 0.35 m/s-0.83 m/s; average of 0.51 m/s (SD 0.14 m/s) and 15 A-delta afferents conducted within somewhat faster range of 1.2 m/s-7.9 m/s; average 2.62 m/s (SD 2.16 m/s). Responses of afferent neurons were intensity dependent; the number of action potentials recorded in response to increases in laser intensity of both pulse types incremented essentially linearly (Figure 3).

### Selective Activation by Long or Short Diode Laser Pulses

**Figure 4:**
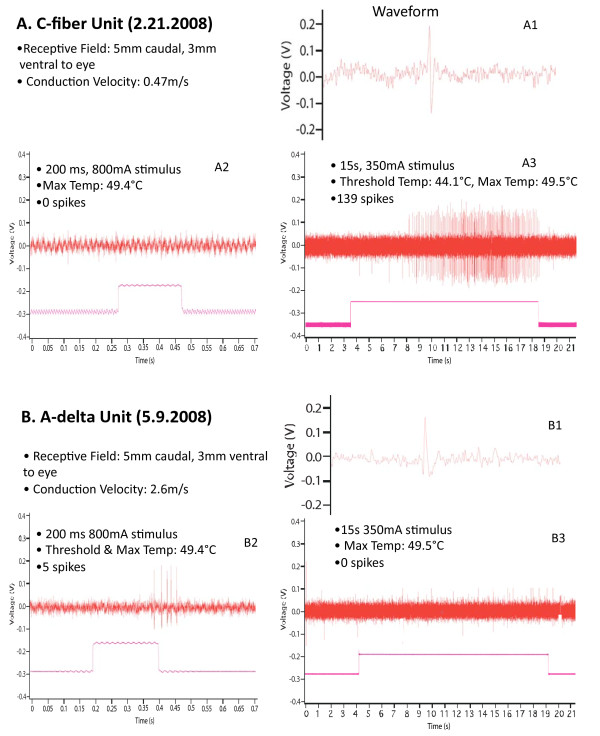
**Examples of responses of individual C fiber and A-delta nociceptor units to long or short laser pulses**. A. Response of a C fiber nociceptor, A1 - action potential shape; A2 - lack of response to short pulse; A3 - response to long pulse; B. Response of an A-delta nociceptor, B1 - action potential shape; B2 - response to short pulse; B3 - lack of response to long pulse.

Of the 19 units in the slowest group, 15 responded only to the long pulse (LP), slow heating rate laser stimulation but not to the short pulse (SP), high heating rate laser stimulation, and 4 responded to both LP and SP (Figure 2). Of the 15 units in the faster conducting group, 10 responded only to SP but not to LP, 2 responded to LP but not to SP, and 3 responded to both LP and SP. Figure 4 illustrates examples of units in the C fiber conduction velocity range (4a) and A-delta conduction range (4b), that responded only to long pulse or short pulse respectively. The 2 units that responded to LP only fell into the faster group exhibited CVs of 1.25 m/s and 1.36 m/s, which is the lowest end of that CV range, near the borderline of the two populations. Of 34 total units, 7 responded to both LP and SP laser stimulation. These units fell into both nociceptor conduction ranges, exhibiting CVs in the range of 0.63 m/s - 3.33 m/s.

### Skin Temperature Measurements

**Figure 5:**
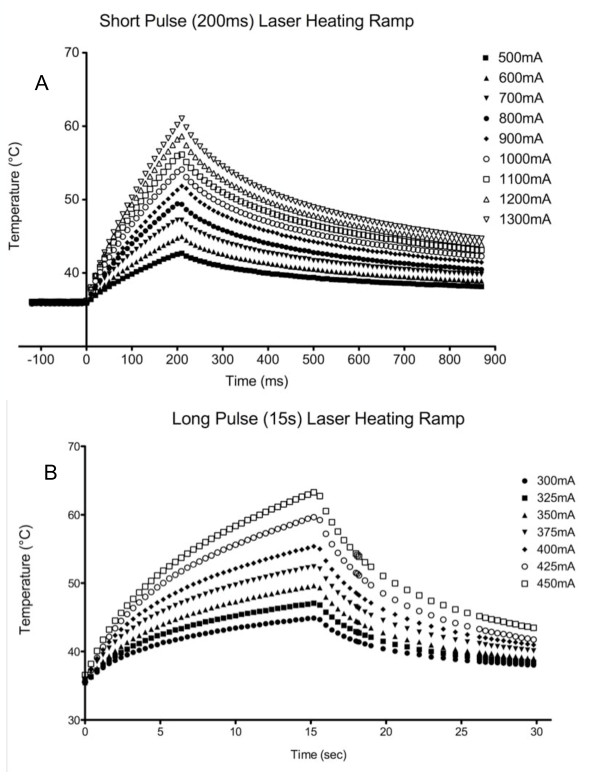
**Skin Temperature Changes in Response to Laser Diode Stimulation**. Surface skin temperature was measured using a high-speed thermal camera, and showed a decelerating increase in temperature produced by the constant intensity stimulus. This observation was true both for short, high power and long, lower power laser diode stimuli.

**Figure 6:**
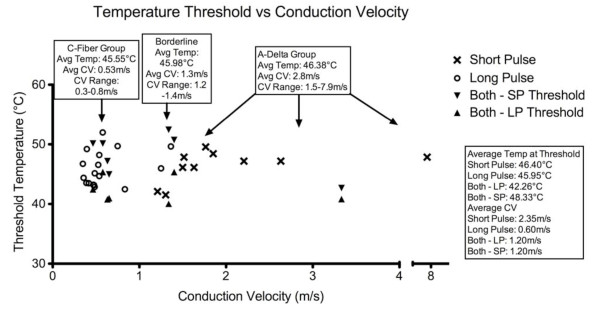
**Relationship Between Threshold Temperature and Conduction Velocity**. Temperature response thresholds were in a similar range for neurons that responded to either long or short pulses, however, all neurons that responded to both pulse types responded at lower temperatures to the long pulse.

To further characterize trigeminal cutaneous nociceptors, in some experiments, skin temperature was measured with a thermal camera during laser stimulation to identify the temperature threshold of laser-elicited responses. Figure 5 demonstrates that skin temperature increased in a decelerating function with peak temperatures increasing with increasing laser power, which is similar to that we observed for constant intensity projector bulb emitted radiant heat [35]. Based on these measurements, temperatures were calculated for all other laser-evoked responses. To better distinguish the nociceptor populations, a third group (labeled "Borderline") was used to describe units that fired within a CV range of 1.2-1.4 m/s (See fig 6). The calculated average (± SEM) surface skin temperatures at nociceptor activation threshold of the C-fiber, "Borderline," and A-delta groups were 45.55°C (± 0.66), 45.98°C (± 1.63), and 46.38°C (± 0.85), respectively. The calculated average (± SEM) temperatures at threshold for units that responded selectively to LP or SP were similar at 45.95°C (± 0.70) and 46.40°C (± 0.83), respectively. For units that responded to both heating rates, the average (± SEM) temperature at threshold was 42.26°C (± 0.84) for LP and 48.33°C (± 1.32) for SP.

## Discussion

The trigeminal nervous system is integral in numerous pain states that are unique to that system, including various types of headache, temporomandibular joint disease, dental pain, and others. Yet, understanding of this system is relatively minimal. Investigations into the thermal response properties of sub-cranial nociceptors have been instrumental in defining the potential efficacy of putative analgesic therapies. Although treatments for trigeminal pain overlap those for somatic pain, they can be quite distinct (e.g., triptan drugs for migraine headache). Work described in this paper provides some of the first *in vivo *characterization of the thermal responsivity of trigeminal nociceptors.

Since the original publication of the differential sensitivity of C versus A-delta thermonociceptors to different radiant heating rates [36,37], this distinction has been used in over 300 studies. However, stimulus control and repeatability is inherently limited by such factors as the broad spectrum of applied wavelengths and the dependence of power conversion on filament temperature for typical light sources, such as projection bulbs. We have previously shown an infrared diode laser stimulator to be reproducibly nociceptor-selective [38]. Our electrophysiological data demonstrate that selectivity of the infrared diode laser is consistent with these previous behavioral data and that the threshold temperatures are consistent with the results of our experiments using the same laser to activate TRPV1 channels on dissociated dorsal root ganglia neurons and HEK cells [39,40].

The results of the current work demonstrate that there are two distinct, but overlapping populations of thermally sensitive nociceptors terminating in trigeminally-innervated skin. The first population (n = 19) responds primarily or exclusively to slow heating ramps, and is undoubtedly very small diameter and unmyelinated (C fibers) as they are remarkably slowly conducting (average CV 0.51 +/- 0.03 m/s (SEM)). The second population (n = 15), which respond predominantly or exclusively to fast heating rates, were likely small diameter and either unmyelinated (faster C) or thinly myelinated (A-delta) fibers as they conducted at somewhat higher rates (average CV 2.1 m/s +/- 0.44 m/s (SEM)). Interestingly, this separation of afferent populations is qualitatively similar, but quantitatively shifted from thermonociceptors innervating the hindpaw [37]. In this study, we found that slow heating ramps selectively activated a population of afferent neurons (likely C fibers) with an average CV of 0.92 m/s (range: 0.6 to 2.3 m/s), whereas rapid heating selectively activated a faster conducting population with an average CV of 8.9 m/s (range: 3.0 to 17.2 m/s). Note that the slowest conducting afferent recorded in this group was faster than the average rate for the trigeminal recordings. Thus, our data are consistent with Levy and Strassman's statement that "trigeminal primary afferents may have somewhat slower CVs than their spinal analogs" [41]. Response temperature thresholds were also qualitatively similar but quantitatively different from those of paw afferents. In our previous experiment (using a projector bulb heat source), surface temperature threshold for C fibers was 43.8°C (+/- SEM 0.4) and almost identical 43.9°C (+/- SEM 0.3) for A-delta nociceptors. Temperatures recorded for trigeminal afferents were also similar for the fast C and slow A-delta group: 45.95°C (+/- 0.67 SEM) and 46.40°C (+/- 0.31 SEM), respectively. Thus, while the thresholds were similar between the groups within a system (trigeminal, somatic), there are dissimilarities between them. These results again point out that the trigeminal thermonociceptive system is unique.

The distinct properties of trigeminal thermonociceptive afferents, when compared to those of the body suggest that nociceptors from these different areas might provide differentiable sensory functions. The relatively slow conduction of the faster (fast C/slow A-delta) group might be consistent with the relatively short conduction distances of the head when compared to the limbs. However, rat paraspinous unmyelinated afferents, which have very short conduction distances have an average CV of 0.84 m/s (SD = 0.26), which is similar to that observed for the hindpaws, and substantially faster than trigeminal CVs. These data suggest that it may not simply be a matter of conduction distance that predicts the slow conduction of trigeminal afferents. Preliminary psychophysical results suggest, in fact that the quality of sensation produced by activation of trigeminal nociceptors may be dissimilar to that produced by activation of somatic nociceptors. While intense, brief laser pulses applied to the fingers, arms or toes always produce "pricking" pain[39,42], the same stimulus applied to the face never does. In fact the sensation produced is described as "hot-sharp" - a distinction that might be related to the relatively slow conduction velocity of the second, "faster" group of trigeminal nociceptors.

## Conclusions

The results of these experiments provide the first characterization of trigeminal thermonociceptive afferent neurons in rats. In particular, the careful measurement of temperature thresholds and action potential conduction velocities afford an understanding of the thermal response properties of nociceptors innervating the skin. These results indicate that there are at least two populations of trigeminal thermonociceptors, which are differentially activated by high or low rates of skin heating and which have differentiable conduction velocities. These results also point to clear differences between trigeminal and somatic nociceptors that had not previously been observed including higher average temperature thresholds and overall slower conduction velocities. Observation of these differences was made possible by the use of highly controlled laser diode thermal stimulation. These distinctions may be important in elucidating the mechanisms of trigeminal pain and in developing novel therapies for the treatment of the myriad of pain states mediated by the trigeminal system. For example we have previously demonstrated that, for somatic pain, C fiber and A-delta nociceptor mediated responses are differentially attenuated by mu, delta1 and delta2 opiate receptors which may indicate preferential effects for different types of clinical pain[11,43]. Consistent with this idea, Scherrer et al.[44] recently demonstrated preferential analgesic effects of mu opioids for (likely C fiber mediated) thermal nociception and delta opioids for (likely A-delta fiber mediated) mechanical nociception. Thus, a clear understanding of and the capacity to differentially activate C or A-delta trigeminal nociceptors may prove useful in examining the potential utility of putative analgesic therapeutics for trigeminal pain syndromes.

## Methods

### Animals

30 male Sprague-Dawley rats (300-400 grams, Charles-River Laboratories) were used in this study. The animals were housed in a temperature-controlled room (22 ± 1°C) in a 12 hour light/dark cycle with food and water provided *ad libitum*. The experimental procedures were approved by the Stanford University Institutional Animal Care Committee.

### Surgery and skin preparation

Rats were initially anesthetized (isoflurane, 3-4%) in a chamber, then moved to mask anesthesia (isoflurane 2-3%) during surgery. The isoflurane concentration was adjusted as needed so that a strong tail or paw pinch failed to evoke a withdrawal response. A midline skin incision was made over the trachea, the sternohyoid muscles were separated using blunt dissection, a tracheostomy tube (1 cm length, 2.5 mm diameter; Kent Scientific, Torrington, CT) was implanted, a jugular vein was cannulated with polyethylene-50 tubing for the delivery of intravenous fluids during the duration of the experiment and wound clips were used to close the incision. The left side of the rat's head was shaved thoroughly with an electric razor, first with a course attachment followed by a fine attachment. This procedure allowed complete removal of hair in the area of stimulation without causing skin damage or excessive irritation. The rat was then moved to a stereotactic frame, where a midline skin incision was made extending from the intraural line rostrally 2 cm. The fascia overlying the skull at the incision site was scraped clear with a scalpel and a trephination in the skull was performed from 0-5 mm lateral and 0-5 mm caudal to bregma [34]. The dura was left intact.

### Electrophysiology

Core body temperature was monitored rectally and maintained at 37 ± 0.5°C with a heated water circulating system (T/Pump, model TP500, Gaymar, Orchard Park, NY). Anesthesia was maintained by delivery of 1.4-2% isoflurane in a mixture of room air and oxygen (~70% air, 30% oxygen). The animal was ventilated using a pressure-controlled ventilator (TOPO model, Kent Scientific, Torrington, CT). Expired PCO_2 _was monitored by a Datex 254 gas analyzer (Datex-Ohmeda, Tewksbury, MA) and maintained between 30 and 40 mm Hg by adjustment of respiratory rate. Respiratory parameters were: peak inspiratory pressure = 7-10 cm H_2_O, inspiratory flow = 0.6-1.2 L/min, respiratory rate = 60-90 BPM. Rats were paralyzed with pancuronium bromide (1 mg/kg/h). A 0.5 MΩ epoxylate-insulated, platinum-tipped, tungsten microelectrode (FHC# UEWMGGSEBTNM, FHC, Bowdoinham, ME) was used to record extracellular single-unit activity of trigeminal ganglion neurons. The electrode was zeroed at bregma and was then advanced through the dura and the brain, initially at 3 mm lateral and 1.5 mm caudal to bregma, according to previously reported stereotactic coordinates [34,45,46] using a hydraulic microdrive (Kopf Instruments, Tujunga, CA). The electrode was advanced at rates of 12.5-25 μm/sec until reaching 9 mm in depth, and then at 1.25 μm/sec until activity was evoked by facial stimulation, at which time advancement was continued in 0.8-1.0 μm bursts during search stimulation. Search stimulation consisted of innocuous mechanical (cotton-tipped or blunt probe) or constant-current bipolar electrical stimulation (2 mm diameter silver ball electrodes with 2 mm separation; 0.1-2 ms duration, 30-50 V; Grass S48 Stimulator, Quincy, MA) of the ipsilateral facial skin. Response properties of single units isolated using touch and/or electrical stimulation were then characterized using von Frey hair stimulation and the receptive field area (RFA) was noted. Action potentials were amplified (DAM 80, WPI, Sarasota, FL) and recorded using a Powerlab interface and Chart 5 software (AD Instruments, Grand Junction, CO) at sampling rates of 20 kHz. Data was analyzed offline using Chart 5 and Microsoft Excel. At the end of the study, rats were euthanized by anesthetic overdose and exsanguination. The trigeminal ganglion and nerve were extracted from some rats after electrophysiological recording to enable the calculation of conduction velocity.

### Laser Protocol

A diode laser (LASS-10, Lasmed LLC, CA, USA) with a power of 10 W, wavelength of 980 nm, and an adjustable spot diameter between 2 and 5 mm was used. Units that were driven by long-duration (2-10s) mechanical stimuli and responded with an increasingly greater number of action potentials in response to increased stimulus forces into the noxious range, including pinch, were considered nociceptors and were selected for further study. These selected units were then stimulated with the diode laser and positioned with a micromanipulator for fine 3-dimensional control. Units that were determined to conduct at A-fiber velocities (> 1.5 m/s, based on Levy & Strassman [41] with previous electrical stimulation of the face using ball electrodes were stimulated first with short-pulse (2 mm spot diameter, 200 ms stimulus duration, 500 mA - 1400 mA) and then with long-pulse (5 mm spot diameter, 5-15 s duration, 250 mA - 400 mA) laser. Those that conducted at C-fiber velocities (≤1.5 m/s, based on Levy & Strassman [41]) were stimulated first with long-pulse then with short-pulse laser. For units that were not previously stimulated electrically, short-pulse or long-pulse stimulation was performed first in random order. Units that did not respond to laser stimulation were characterized as mechanonociceptors and usually not studied further. Units that responded to laser thermal stimulation were considered mechano-heat nociceptors [47-49] and studied further.

### Thermal Camera Protocol

While some rats were deeply anesthetized, a high spatial resolution (15 pixels per 1 mm) thermal imaging camera (Flir SC6000; Flir, Boston, MA, USA) was positioned over the skin area to be laser stimulated. This camera has a high enough sampling rate (200 Hz) to accurately (0.1°C repeatability) record surface skin temperatures when the high power (high rate) laser pulses were applied. Skin was lazed at various spot sizes, intensities and durations and the skin temperature changes recorded. Temperature changes were highly consistent across animals and so temperature changes for other animals were calculated from measured temperatures in these rats.

## Competing interests

MIN is the CEO of Lasmed, LLC., which provided the laser and initial stimulus protocol for this work. DCY is a co-owner of a patent held by Lasmed describing the use of diode lasers in pain research.

## Authors' contributions

JMC established the electrophysiological methods used and contributed to the writing of the manuscript; NAM carried out further electrophysiological experiments and contributed to the analysis of the data and manuscript presentation of that data; MK carried out the thermal camera experiments and contributed to the preparation of the portion of the manuscript devoted to that work; MIN developed the laser stimulation device and protocol, contributed to the temperature-response analysis and that portion of the manuscript; DCY provided overall direction, helped to develop the experiments, and contributed to the preparation of the manuscript.

All authors have read and approved the final manuscript

## Author Information

In some ways, this paper is a follow-on to a series of papers published by DCY in the mid 1990's, describing differential thermal activation of somatic nociceptors in rats, monkeys and man.
